# Functional fitness benchmark values for older adults: a systematic review

**DOI:** 10.3389/fpubh.2024.1335311

**Published:** 2024-03-21

**Authors:** Marco Cossio-Bolaños, Ruben Vidal-Espinoza, Ignacio Villar-Cifuentes, Luis Felipe Castelli Correia de Campos, Marcela Silva Ramos de Lázari, Camilo Urra-Albornoz, Jose Sulla-Torres, Rossana Gomez-Campos

**Affiliations:** ^1^Departamento de Ciencias de la Actividad Física, Universidad Católica del Maule, Talca, Chile; ^2^Faculty of Education, Psychology and Sport Sciences, University of Huelva, Huelva, Spain; ^3^Universidad Católica Silva Henríquez, Santiago, Chile; ^4^Universidad del Bio Bio, Chillán, Chile; ^5^Núcleo de Investigación en Ciencias de la Motricidad Humana, Universidad Adventista de Chile, Chillán, Chile; ^6^Faculdade de Ciências Médicas, Universidade Estadual de Campinas, São Paulo, Brazil; ^7^Universidad Católica de Santa María, Arequipa, Peru

**Keywords:** percentiles, senior fitness test, functional fitness, older adult, physical fitness

## Abstract

**Introduction:**

The use of normative values and/or standards of functional fitness in adults is relevant to overall health and well-being. The objectives of the study were: to identify the physical tests of the senior fitness test (SFT) that have been applied since its proposal and to describe the proposed percentiles according to age, sex and country.

**Methods:**

A systematic review study was conducted in the Pubmed and Scopus databases. As eligibility criteria, we considered the period from 1999 to 2022 that presented data on SFT test used in the population over 60  years of age and that described normative values through percentiles. MeSH were used as: (1) Physical fitness, Exercise test, Senior Fitness Test, Functional fitness, Cardiorespiratory fitness, (2) older adult, aged, (3) Reference standards, standards, standards of care. Boolean operators “AND” and “OR” were included. Data extracted from the selected studies included: year of publication, country, sample age, sample size, sample sex, fitness component.

**Results and discussion:**

Seven studies were identified in five countries (03 in China, 01 in Poland, 01 in Portugal, 01 in Spain and 01 in United States). The age range ranged from 60 to 103  years. The studies were conducted in both sexes. The study with the smallest sample size was by Chung et al. (China) with 944 participants and the largest number of participants was the study by Rikli and Jones in the United States with 7,183 participants. In general, no study was able to complete 100% (8 components) of the tests proposed in the SFT. Normative values were presented through percentile distribution (p10, p50 and p90) organized by age ranges. Males presented better performance in FPF tests than females in all tests. Since the first publication of the SFT until 2022, seven articles have been published in countries such as United States, China (three regional studies), Poland, Portugal and Spain. No study has published the complete battery with its eight components. The percentiles of functional fitness reflect decline with advancing age.

**Systematic review registration:**

PROSPERO (CRD42023441294: https://www.crd.york.ac.uk/prospero/display_record.php?ID=CRD42023441294).

## Introduction

Aging in general is associated with declining physical function that affects vital processes that are critical for functional independence, social engagement, and quality of life (1). Indeed, as one ages, physical fitness gradually declines, so older adults (OAs) face risks of functional decline and frailty, resulting in loss of independence in activities of daily living (2).

Functional fitness (FF) is defined as the physical capacity to perform daily activities safely, independently and without excessive fatigue (3). So its assessment in older people is extremely important, especially when it comes to healthy aging and the control of healthcare costs (4).

Functional fitness levels in OA are the result of dynamic socioeconomic changes, as well as various living conditions, socio-cultural, ethnic, genetic ethnicity, genetics, nutrition, geography, and physical activity levels (5, 6).

In recent years, several studies have shown that lack of physical activity, along with non-communicable diseases and lifestyle (7), lead to a decrease in daily mobility and functional capacity (6, 8).

In that sense, it is important to assess functional fitness as it is part of the basis of overall health and well-being and is more easily understood by examining its components or parts (9). For example, the main components of functional fitness are lower and upper body strength, lower and upper body flexibility, aerobic fitness and motor agility/dynamic balance (4). Even the morphological component (Anthropometric indices and body composition) is considered, since older adults should maintain a healthy weight and body composition during aging (10).

The assessment of functional fitness in OAs serves to estimate and track the rate of its decline in individual tests with the course of age (3). For this purpose, normative and/or standard values according to age and sex are necessary.

In fact, more than 24 years ago, Rikli and Jones (3), developed a standard tool (Test battery) for older adults aged 60 years or more, called senior fitness test (SFT). It describes eight tests: Body Mass Index (BMI); push-up and stand and sit in a chair for 30 s, which measures upper and lower body strength; the stretch and back scratch test to measure upper body flexibility; the 6-min walk and 2-min step test to measure aerobic endurance; and the 8-foot test to measure dynamic balance.

This battery, has been used in recent years, in several studies worldwide (4, 11–13), whose proposals have been specifically designed taking into account the unique functional needs to perform daily activities (9). In addition, the genetic and ethnic heterogeneity of OAs, as well as cross-cultural differences, have been taken into consideration to construct normative values of SFT among OAs of the same age (4).

Consequently, given the existence of multiple studies that have proposed normative SFT values in OAs in various parts of the world, to our knowledge there is no systematic review that has identified the SFT tests that have been used in OAs. Not even the percentiles of each of these tests have been described. In fact, this information may be relevant to practitioners and researchers working with OA within the community. Furthermore, the use and application of percentiles in various sociocultural settings may serve to compare functional fitness across populations and to understand rates of loss of functional independence across countries.

Therefore, the objectives of this study were: To identify the physical tests of the senior fitness test (SFT) that have been applied since its proposal and to describe the proposed percentiles as a function of age, sex and country.

## Methods

### Type of study

This systematic review was conducted and reported according to the Preferred Reporting Items for Systematic Reviews and Meta-Analyses (PRISMA) guidelines (14, 15) and it is registered in the PROSPERO database under the number CRD42023441294.

The PICO strategy was used to formulate the northern question, composed of the following elements: P (Population: older adults); I (Intervention: application of SFT tests) and O (Outcomes: proposed normative SFT values). Comparison (C) between the normative values proposed in the studies was not performed. Thus, the following PICO question is formulated: What are the most frequently documented individual tests in the scientific literature related to functional fitness in older adults and to what extent are these tests described by percentile distributions?”

As eligibility criteria, published articles from primary sources presenting data on SFT tests used in the adult population over 60 years of age and presenting normative values through percentiles were considered.

### Sources of information and search strategy

Research data were extracted from the US National Library of Medicine’s PubMed database^1^ and Scopus. These databases considers biomedical and life sciences literature. The search period was between December 1 and December 31, 2022. Articles published between 1999 and 2022 were considered.

The main search terms were defined through group discussions among the research team and considered: Senior fitness test, functional fitness, older adult and percentiles. MeSH was used to obtain relevant terms in PubMed and Scopus such as: (1) Physical fitness, Exercise test, Functional fitness, Functional fitness, Senior Fitness test, Cardiorespiratory fitness, (2) older adult, aged, (3) Reference standards, percentiles, standards, standards of care. The terms were used in isolation and in combination, considering the Boolean operators “AND” and “OR” to order them. They were grouped into two or three, and a new search was performed. These terms were searched for in the title, abstract and keywords of the manuscripts. In addition, a manual search was performed for articles that were not in the database, e.g., studies cited by other manuscripts. Search filters were applied to eliminate case reports of population with physical disabilities and systematic reviews. The search was limited to articles investigating adults older than 60 years.

In recent years worldwide the population of older adults is ostensibly increasing, so it is estimated that by 2050 the number of adults over 60 years of age will almost double, representing more than 21% of the world’s population (16). So, studying functional fitness in over-60s is critical to address the health and wellness needs of this growing population. It is considered a key indicator of a person’s ability to carry out daily activities independently and maintain a good quality of life.

### Inclusion and exclusion criteria

Studies were eligible if they met the following inclusion criteria: (1) participants were adults older than 60 years; (2) applied the SFTs; (3) presented normative values expressed as percentiles of the SFTs as outcomes. Studies were excluded if (1) the publication was a systematic review, abstract, study protocol, letter, commentary, study reporting qualitative data, dissertation, or poster abstract; (2) the work was published before 1999; (3) applied scales or questionnaires, which assessed indirectly or by interview.

It should be noted that only methods/evaluations that directly assess performance on pre-established tests are taken into account.

The following is a description of each of the SFT tests according to Rikli and Jones (3):

**Push-ups in 30 s (repetitions):** measures arm strength and endurance by counting the number of push-ups performed correctly in 30 s. This requires women to repeatedly lift a weight of 2.27 kg (5 lb) and men a weight of 3.63 kg (8 lb) for 30 s.

**Standing Chair (reps) in 30 s:** Reflects lower body strength. Requires individuals to stand and sit in a chair for 30 s. The number of repetitions is recorded.

**Sit and stretch in a chair (cm):** Aims to assess lower body flexibility. The distance reached in centimeters is recorded.

**Scratching the back (cm):** The objective is to measure the flexibility of the upper body. The distance is measured and recorded in centimeters.

**8-ft up-and-go (sec):** Evaluates agility and dynamic balance. The test begins seated in a chair and must travel 2.45 m and reach the starting position. Time is recorded in seconds.

**2-min step test (rep):** Evaluates aerobic endurance. The maximum number of knee lifts performed in 2 min is counted. During the stationary gait, a midpoint between the patella and the anterior superior iliac spine must be produced. The number of repetitions of the right knee is counted.

**6-min walk (m):** Its objective is to evaluate aerobic endurance by walking in meters. If it is not possible to perform the 6-min walk test, this test can be substituted by the 2-min walk test.

### Selection process

After the literature search, an initial selection of studies was made according to the reading of the title and abstract. One reviewer (MC) performed both steps to identify those studies that met the inclusion criteria. Next, another reviewer (RG) checked for agreement. In the case of discrepancies, they were resolved with the review of the full text by the research group.

In the next step, a full-text analysis was performed to verify whether the studies met the inclusion criteria. Subsequently, the research group conducted a manual search for other studies that had not yet been found in the initial search. Finally, full-text analysis and data extraction of the selected studies was performed.

### Data extraction

Data extracted from the selected studies included: year of publication, country, sample age, sample size, sample sex, SFT test. The collected data were organized in Microsoft Excel spreadsheets. The methodological quality of the eligible studies in this review was evaluated according to the Physiotherapy Evidence Database (PEDro Scale) (17). The PEDro Scale helps to identify, by means of 11 evaluation criteria, which of the studies may have internal validity (criteria 2 to 9). As well as containing sufficient statistical information so that their results can be interpreted (criteria 10 and 11). After applying the scale, the studies were classified on the basis of the scores obtained as follows: those with scores between zero and four were considered of low quality; four to five were considered of moderate quality; six to eight were considered of high quality; and 9 to 10 were considered of excellent methodological quality (17).

## Results

### Selection process

**Figure 1:**
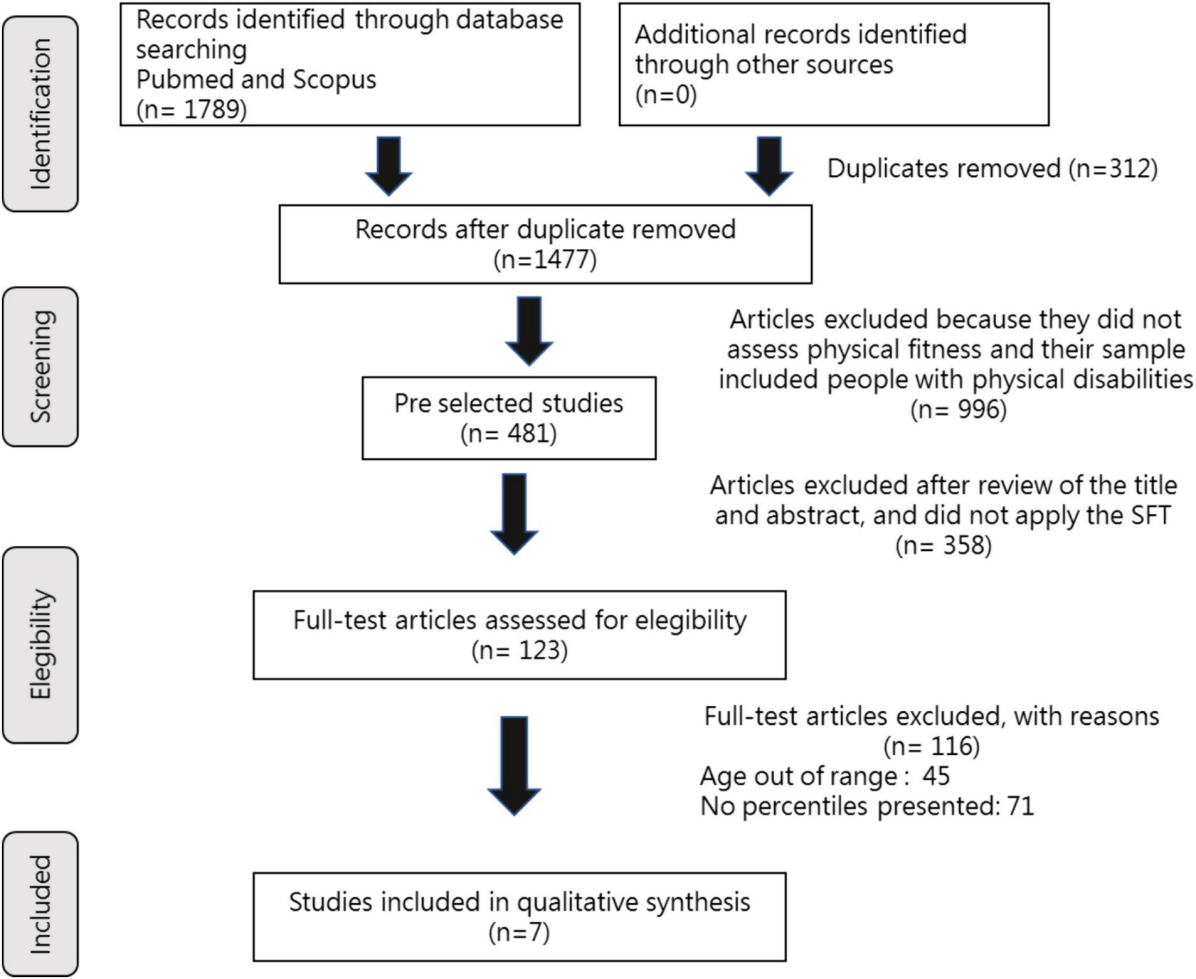
Prisma flow diagram showing the summary of the literature searches.

Of the total 1789 studies, 1,308 were excluded because they did not assess physical fitness. Of the remaining 481, 358 were excluded because they did not apply the SFT tests. Next, the full text of the 123 was analyzed. At this stage, 116 additional articles were excluded, resulting in a total of 7 articles that were included in the review. The process and outcome of the literature selection is presented in detail in Figure 1.

In the evaluation of methodological quality, 14% (*n* = 1) of the studies were considered to be of moderate quality, obtaining 5 points. The other 86% (*n* = 6) were of high quality. Of these, three obtained 6 points and another three obtained 7 points, as shown in Table 1.

**Table 1:** Indicators of systematized articles.

Authors	Country	Gender	Sample	60–64 y	65–69 y	70–74 y	75–79 y	80–84 y	>85 y	Total	Methodological quality, Pedro scale (score)
Xu et al. (18)	Suzhuo Region (China)	Females	Probabilistic	NS	NS	NS	NS	NS	NS	664	6
		Males		NS	NS	NS	NS	NS	NS	458	
Zhao et al. (13)	Nanjing Region (China)	Females	Not probabilistic	~269	~296	~235	~201	~149	~52	1,270	6
		Males		~220	~236	~217	~209	~164	~39	1,128	
Ignasiak et al. (19)	Polonia	Females	Not probabilistic	1,165	1,189	746	488	379	127	4,164	5
		Males		291	320	260	176	82	36	1,203	
Chung et al. (20)	China	Females	Not probabilistic	--	136	124	144	118	--	522	7
		Males		--	112	100	107	103	--	422	
Marques et al. (4)	Portugal	Females	Not probabilistic	--	839	793	589	419	351	3,121	7
		Males		--	416	367	280	238	204	1,591	
Gusi et al. (11)	Spain	Females	Not probabilistic	1,351	1,516	1,401	787	262	75	5,610	7
		Males		99	217	230	154	72	31	839	
Rikli and Jones (3)	USA	Females	Not probabilistic	620	1,084	1,298	987	543	512	5,048	6
		Males		241	482	515	464	241	192	2,135	

~, Approximately; NS, not specified; PEDro, Physiotherapy Evidence Database.

The indicators characterizing the systematized studies are shown in Table 1. Seven studies were identified that were carried out between 1999 and 2022 in five countries (03 in China, 01 in Poland, 01 in Portugal, 01 in Spain and 01 in the United States), with an age range from 60 to 103 years. All the studies were conducted in both sexes, where the smallest sample size was reported in the study by Chung et al. (China) (20) with 944 participants and the largest number of participants was observed in the study conducted by Rikli and Jones (3) in United States with 7,183 participants.

Table 2 shows the eight components used in the SFT. The studies by Xu et al. (18), Zhao et al. (13) and Chung et al. (20) have evaluated seven tests, except the 6-min walk test, while the study by Marques et al. (4) only did not evaluate the 2-min step test. On the other hand, the study by Ignasiak et al. (19) did not use the tests having to do with BMI and the 6-min walk test. The study by Gusi et al. (11) did not use three tests (30-s Arm curl, 30-s chair stand and 2-min step test). Studies that have evaluated the 30-s Arm curl (rep) test have used the original weight proposed by Rikli and Jons (3). For men 3.63 kg (8 lbs) and for women 2.27 kg (5 lbs). Overall, no study reached 100% completion of the tests proposed in the original study by Rikli and Jones (3). In addition, only one study by Xu et al. (18) in the Suzhuo Region (China) used probability sample selection to propose percentiles. While the other studies used non-probabilistic selection, even the study by Rikli and Jones (3) in the United States used non-probabilistic selection.

**Table 2:** Components used by the systematized studies.

n°	Test	Xu et al.	Zhao et al.	Ignasiak et al.	Chung et al.	Marques et al.	Gusi et al.	Rikli and Jones
1	BMI (kg/m2)	√	√	Χ	√	√	√	√
2	30-s push-up (reps)	√	√	√	√	√	Χ	√
3	30-s standing chair (rep)	√	√	√	√	√	Χ	√
4	Sit and stretch in chair (cm)	√	√	√	√	√	√	√
5	Back scratch (cm)	√	√	√	√	√	√	√
6	8-ft up-and-go (sec)	√	√	√	√	√	√	√
7	2-min step test (rep)	√	√	Χ	√	Χ	Χ	√
8	6-min walk (m)	Χ	Χ	√	Χ	√	√	√
	Total	7	7	6	7	7	5	8

The percentile distribution (p10, p50 and p90) of BMI of the systematized studies are shown in Table 3. Of the seven studies, six investigations reported BMI percentiles in both sexes, only the study by Ignasiak et al. (19) conducted in Poland did not show percentiles. The percentiles were distributed according to age ranges (60–64 years, 65–69 years and ending in 90–94 years).

**Table 3:** Body mass index (BMI) percentiles of systematized studies in male and female OAs.

Age group	Xu et al.			Zhao et al.			Ignasiak et al.			Chung et al.			Marques et al.			Gusi et.al			Rikli and Jones		
	p10	p50	p90	p10	p50	p90	p10	p50	p90	p10	p50	p90	p10	p50	p90	p10	p50	p90	p10	p50	p90
Males																					
60–64	21.3	26.3	28	21.9	25.1	28.5	--	--	--	--	--	--	--	--	--	24.5	30	36.7	22	27.4	32.8
65–69	20.3	23.9	27.8	21	24.8	28.9	--	--	--	20.3	23.6	28.3	23.1	27.6	32.3	24.8	29.4	34.9	22.1	27.5	32.9
70–74	20.1	25	28.6	21.2	25	29.3	--	--	--	21.0	24.6	28.9	22.9	27.4	32.4	25.1	29.3	34.6	21.6	26.6	31.6
75–79	20.2	24.7	27.8	20.5	24.8	29.5	--	--	--	20.3	24.5	29.1	22.6	27.2	32.4	25.7	29.5	33.5	21.4	26.4	31.4
80–84	20.7	24.4	27.4	20.4	24.2	27.6	--	--	--	20.1	23.4	27.4	22.5	27.1	32.6	24.9	28.5	34.6	21.7	26.1	30.5
>85	--	--	--	20.1	24.3	29.3	--	--	--	--	--	--	22	26.4	31.9	24.1	28.2	33.1	21.8	24.9	28
Females																					
60–64	20	23.6	27.7	21.9	24.2	28.5	--	--	--	20.2	24	29.1	23.1	--	34.8	25.5	30.7	37.1	19.6	26.3	33
65–69	20.2	24	27.7	20.7	24.7	29.3	--	--	--	19.5	23.6	28.6	23	28.2	34.1	25.6	30.7	37.1	19.8	26.5	33.2
70–74	20	23.6	27.6	20.2	24.5	29.8	--	--	--	19.3	23.8	26.4	22.8	27.9	33.9	25.6	30.5	36.6	20.3	26.1	31.9
75–79	18.7	23.1	28	19.8	24.3	29.6	--	--	--	19.5	23.6	29.4	22.7	27.8	34.1	25.1	30.4	25.9	19.8	25.4	31
80–84	19.3	22.9	26	19.6	23.5	29.2	--	--	--	--	--	--	21.9	27.8	33.3	24.9	30.1	35.6	19.6	24.7	30
>85	--	--	--	18.4	24	30.6	--	--	--	--	--	--	--	27	--	22.7	28.7	35	19.5	24.3	29

During these age categories it is observed that the three studies conducted in China by Xu et al. (18), Zhao et al. (13) and Chung et al. (20) evidenced lower BMI values in both sexes in relation to the other studies. In addition, the study conducted by Gusi et al. (11) in Spain, presents higher BMI percentile values than the other studies in both sexes. These values are higher in men from ~4.8 to 6.4 kg/m2 and in women from ~6.7 to 7.1 kg/m2.

The percentile values of the SFT tests for both sexes are shown in Tables 3, 4. All seven studies describe the p10, p50 and p90 percentiles for all physical tests, except the studies performed in China by Xu et al. (18), Zhao et al. (13) and Chung et al. (20) did not report percentiles for the 6 min walk test. In addition, the study by Gusi et al. (11) performed in Spain did not report percentile data for Arm curl and 30-s chair stand. The other studies reported all tests. It is also highlighted that men presented better performance in the FPF tests than females in all tests (Table 5).

**Table 4:** Percentiles of functional physical fitness of systematized studies in female OA.

Age group	Xu et al.			Zhao et al			Ignasiak et al.			Chung et al.			Marques et al.			Gusi et al.			Rikli and Jones		
	p10	p50	p90	p10	p50	p90	p10	p50	p90	p10	p50	p90	p10	p50	p90	p10	p50	p90	p10	p50	p90
6-min walk (m)																					
60–64	--	--	--	--	--	--	351	500	630	--	--	--	--	--	--	320	420	560	495	605	710
65–69	--	--	--	--	--	--	330	477	602	--	--	--	300	510	605	310	410	561	440	570	695
70–74	--	--	--	--	--	--	310	446	567	--	--	--	270	480	580	290	380	512	420	550	675
75–79	--	--	--	--	--	--	284	405	556	--	--	--	173	400	545	250	360	500	365	510	655
80–84	--	--	--	--	--	--	215	376	502	--	--	--	118	300	500	205	320	470	310	460	610
>85	--	--	--	--	--	--	150	300	480	--	--	--	89	225	430	159	280	452	260	425	595
30-s Arm curl (rep)																					
60–64	16	19	23	16	20	26	13	18	26	--	--	--	--	--	--	--	--	--	10	16	22
65–69	15	19	22	9	17	24	12	18	25	7	12	17	11	18	25	--	--	--	10	15	21
70–74	13	17	21	9	16	22	12	17	24	7	13	20	11	17	24	--	--	--	9	15	20
75–79	13	16	20	8	15	21	10	15	22	6	13	18	8	15	22	--	--	--	8	14	20
80–84	10	16	20	6	14	19	9	15	20	5	12	17.2	5	12	20	--	--	--	8	13	18
>85				7	13	19	8	12	16	--	--	--	4	11	19	--	--	--	7	12	17
30-s chair stand (rep)																					
60–64	14	17	22	12	17	21	10	15	21	--	--	--	--	--	--	--	--	--	9	15	20
65–69	12	16	19	11	16	22	10	15	20	10.7	15	21	9	15	21	--	--	--	9	14	18
70–74	10	15	20	11	15	21	10	14	20	11	15.5	21	9	15	21	--	--	--	8	13	18
75–79	10	13	18	8	14	20	9	13	19	8	14	20	6	13	18	--	--	--	7	12	17
80–84	9	13	18	8	13	17	8	13	17	8.4	13	17	3	10	16	--	--	--	6	11	16
>85	--	--	--	7	12	17	7	12	16	--	--	--	2	9	16	--	--	--	5	10	15
Chair sit-and-reach (cm)																					
60–64	-8	4	17.1	-6	6	18	-5	4	17	--	--	--	--	--	--	−11	2	16	−3	2	7
65–69	−6.4	3.5	12.4	−5	6	18	−6	3	16	−7	6	20	−18	0	6	−13	1	16	−3	2	6.5
70–74	−10.1	2	14.6	−6.9	4.8	16.7	−7	2	14	−8	6.5	22.8	−16	−1	4	−13	1	14	−3.5	1.5	6
75–79	−13.8	0.5	11.8	−14	3	14.5	−7	2	13	−15	4	18	−20	−2	3	−15	0	14.9	−4	1	5.5
80–84	−13.1	0	10.7	−14	1	11	−9.5	2	12	−13	1	14	−30	−10	1.2	−16	0	12	−4.5	0.5	5
>85	--	--	--	−15	−2	8	−16	0	12	--	--	--	−30	−13	−2.3	−21	0	9	−4.5	−0.5	4.5
Back scratch (cm)																					
60–64	−15	1	7	−19	0.5	7	−14	0	7	--	--	--	--	--	--	−23	−7	3	−5.5	−5	5
65–69	−17	−1	6	−22	−4	6	−17	−1	6	−14	0.25	6.35	−24	−10	2	−24	−9	2	−6	−1	3.5
70–74	−19	−4.5	5.6	−24	−7	6	−17	−2	6	−15	1	7.9	−29	−11	1	−27	−10	2	−6.5	−1.5	3
75–79	−23	−8	5.8	−27	−9	5.1	−20	−4	5	−18	−0.5	6	−37	−15	0.4	−29	−12	1	−7.5	−2	3
80–84	−22	−7	5	−27	−9	3	−22	−4	4	−20	−2.5	4.9	−46	−21	−2	−33	−16	0	−8	−2.5	2.5
>85	--	--	--	−35	−11	3.4	−25	−10	−2	--	--	--	−45	−23	−6	−35	−17	2	−10	−4	2
8-ft up-and-go (sec)																					
60–64	6.3	5.4	4.6	4.1	5	6.6	4.4	5.7	8	--	--	--	--	--	--	5.61	7.2	10	6.7	5.2	3.7
65–69	7	5.7	5.1	4.4	5.6	7.6	4.7	5.9	8.2	4.62	5.97	7.42	9.1	5.6	4.5	5.8	7.6	10.9	7.1	5.6	4.1
70–74	7.9	6.4	5.3	4.8	5.9	8.4	4.9	6.3	8.8	4.83	5.86	7.93	11.6	6	4.7	6.3	8	12	8	6	4
75–79	8.6	6.7	5.3	5.1	6.8	10.3	5.3	7	10.5	5.2	6.66	10.3	18.3	7.3	5.1	6.5	8.63	13.5	8.3	6.3	4.3
80–84	10.5	6.9	5.7	5.6	7.9	11.6	5.3	7.3	11	5.5	7.5	11.2	23.4	10.6	6	7	10	16.7	10	7.2	4.4
>85	--	--	--	6.2	9.2	14.9	5.9	8.2	11.9	--	--	--	29	12.6	6.4	7	12.4	21.1	11.1	7.9	5.1
2-min step test (rep)																					
60–64	76	96	113	83	106	124	--	--	--	--	--	--	--	--	--	--	--	--	60	91	122
65–69	71	93	111	65	96	116	--	--	--	51	82	107	--	--	--	--	--	--	57	90	123
70–74	63	85	106	64	94	116	--	--	--	58.5	84	113	--	--	--	--	--	--	53	84	116
75–79	61	85	105	59	85	110	--	--	--	53.4	81	104	--	--	--	--	--	--	52	84	115
80–84	52	80	101	49	78	104	--	--	--	49	74	100	--	--	--	--	--	--	46	75	104
>85	--	--	--	40	65	96	--	--	--	--	--	--	--	--	--	--	--	--	42	70	98

P, percentile; Rep, repetitions.

**Table 5:** Percentiles of functional physical fitness of systematized studies in male OAs.

Age group	Xu et al.			Zhao et al.			Ignasiak et al.			Chung et al.			Marques et al.			Gusi et al.			Rikli and Jones		
	p10	p50	p90	p10	p50	p90	p10	p50	p90	p10	p50	p90	p10	p50	p90	p10	p50	p90	p10	p50	p90
6-min walk (m)																					
60–64	--	--	--	--	--	--	420	572	706	--	--	--	--	--	--	315	430	548	555	675	790
65–69	--	--	--	--	--	--	381	559	695	--	--	--	348	568	690	333	420	537	500	630	765
70–74	--	--	--	--	--	--	333	540	694	--	--	--	287	528	660	297	413	520	480	610	745
75–79	--	--	--	--	--	--	280	475	615	--	--	--	208	455	621	260	390	503	395	555	715
80–84	--	--	--	--	--	--	298	450	585	--	--	--	150	355	536	221	370	452	370	525	680
>85	--	--	--	--	--	--	156	304	478	--	--	--	117	295	504	162	305	447	295	475	660
30-s Arm curl (rep)																					
60–64	16	19	23	17	22.5	29	14	20	28	--	--	--	--	--	--	--	--	--	13	19	25
65–69	14	18	22	11	18	26	15	20	27	8	14	21	12	19	26	--	--	--	12	18	25
70–74	13	17	22	8	17	26	13	18	26	6.9	12	18.1	11	18	25	--	--	--	11	17	24
75–79	12	16	20	8	15	23	12	18	24	6	12	17.6	9	16	23	--	--	--	10	16	22
80–84	10	15	18	7	13	19	11	16	23	6	12	16.8	7	14	22	--	--	--	10	16	21
>85	--	--	--	7	13	18	9	14	20	--	--	--	6	13	21	--	--	--	8	14	19
30-s chair stand (rep)																					
60–64	14	17	23	13	17	23	10	16	23	--	--	--	--	--	--	--	--	--	11	16	22
65–69	13	16	22	12	16	24	10	16	23	12	17	24	11	16	23	--	--	--	9	15	21
70–74	11	15	20	10	15	21	10	15	23	10.5	15	20.5	9	15	20	--	--	--	9	15	20
75–79	10	15	18	10	14	20	10	14	22	10	14	20	6	13	19	--	--	--	8	14	19
80–84	9	13	19	9	12	17	9	13	20	9	12.5	17.9	5	12	17	--	--	--	7	12	18
>85	--	--	--	7	12	16	7	11	18	--	--	--	3	11	17	--	--	--	6	11	17
Chair sit-and-reach (cm)																					
60–64	−16	0	13.1	−14	1	11.4	−13	2	14	--	--	--	--	--	--	−17	0	16	−6	0.5	6.5
65–69	−16	0	10.5	−17	1	12	−14	0.5	13	−17	−1	14	−22	−6	3.5	−14	0	15	−6	0	6
70–74	−20	−3	9.1	−19	−1	13	−17	0	12	−20	0	12	−24	−8.5	2.7	−18	−1	12.2	−6.5	−0.5	5.5
75–79	−17	−3	8.1	−20	0	9.7	−14	1	13	−20	−2	10.2	−29	−9	1.9	−24	−4	12	−7	−1	5
80–84	−22	−6	4.7	−25	−8	6.4	−18	0	13	−28	−7.5	8	−30	−14	−1	−26	0	8	−8	−2	4.5
>85	--	--	--	−32	−11	0.9	−21	−1.5	11	--	--	--	−32	−15	−2.6	−27	−12	8	−8	−2.5	3
Back scratch (cm)																					
60–64	−22	−11	3	−27	−12	3.7	−25	−8	6.5	--	--	--	--	--	--	−31	−13	1.4	−10	−3.5	2.5
65–69	−25	−12	5	−27	−10	5	−25	−10	5	−22	−1	8	−34	−15	0	−32	−16	0	−11	−4	2
70–74	−26	−13	2.2	−30	−11	2.3	−27	−10	5.3	−28	−8	6	−38	−17	0	−37	−17	−2	−11	−4.5	2
75–79	−28	−11	4.1	−32	−15	3	−29	−13	2	−33	−12	5.8	−44	−20	−3	−39	−19	−2.2	−12	−5.5	1
80–84	−28	−10	2	−34	−14	4	−30	−15	4	−35	12.5	4	−45	−25	−6	−47	−24	−3	−13	−5.5	1
>85	--	--	--	−42	−25	−7.6	−35	−20	−9	--	--	--	−50	−28	−6.2	−42	−28	−5.4	−13	−6	0
8-ft up-and-go (sec)																					
60–64	6.5	5.7	4.5	4.3	5.4	7.2	4.2	5.3	8	--	--	--	--	--	--	5.34	7.1	10.8	6.4	4.7	3
65–69	7.1	5.8	4.9	4.9	5.3	7.8	4.3	5.5	8.8	3.8	4.84	6.63	7.8	5.1	4	5.39	7.1	10	6.5	5.1	3.8
70–74	8.3	6	5.1	4.3	5.9	8.5	4.4	5.8	9.1	4.31	5.58	7.5	12.3	5.9	4.3	5.7	7.3	10.6	6.8	5.3	3.6
75–79	9.4	6.7	5.1	4.6	6.3	9.5	4.6	6	9.7	4.51	6.1	8.54	16.4	6.9	4.9	6	7.8	12.7	8.3	5.9	3.5
80–84	9.9	7.9	5.7	5.4	7.3	10.6	4.8	7	10.6	5.14	6.99	10.4	18	8.3	5.5	6.53	9.75	16	8.7	6.4	4.1
>85	--	--	--	6	8.8	12.2	4.9	8.2	12	--	--	--	22.8	10.1	5.9	7.1	10.2	21.3	10.5	7.2	3.9
2-min step test (rep)																					
60–64	71	94	112	77	96	116	--	--	--	--	--	--	--	--	--	--	--	--	74	101	128
65–69	69	89	112	69	98	118	--	--	--	61.8	93	119	--	--	--	--	--	--	72	101	130
70–74	62	87	108	68	93	115	--	--	--	67.0	90	111	--	--	--	--	--	--	66	95	125
75–79	65	86	100	64	89	114	--	--	--	61.2	84	110	--	--	--	--	--	--	56	91	125
80–84	55	75	98	48	78	102	--	--	--	43.8	76	101	--	--	--	--	--	--	56	87	118
>85	--	--	--	62	77	104	--	--	--	--	--	--	--	--	--	--	--	--	44	75	106

P, percentile; Rep, repetitions.

## Discussion

The initial objective of this systematic review was to identify the SFT tests that have been used to date. The results have evidenced that out of a total of eight tests that were proposed in the SFT, no study reached 100% of the proposed tests, only 4 studies have completed seven tests, the other studies have reached 6 and 5 tests, respectively.

The 2-min step test and 6-min walk tests were the least frequently used tests in this systematic review. In fact, the 2-min step test, is a motor activity that is based on climbing stairs, so it can be considered more physically demanding in relation to the 6-min walk test that involves walking on a flat and less demanding surface (21). However, both tests demand greater time investment during their evaluation and are relevant for measuring aerobic endurance in OA (22).

The 2-min walk test is useful when a rapid assessment of functional capacity is needed. It has been shown to correlate well with the 6-min walk test in older adults and in people with various chronic diseases (23, 24). Thus, the 2-min walk test can serve as an efficient alternative to longer walk tests, especially in situations where a rapid assessment of functional fitness is needed.

It seems that one of the reasons why the 2-min step test was not evaluated in the studies of Ignasiak et al. (19) conducted in Poland, Marques et al. (4) in Portugal and Gusi et al. (11) in Spain could be related to the higher physical demand as seen above. Similarly, the 6-min walk test was not evaluated by the studies of (13, 18, 20), possibly due to the higher investment time involved in such evaluation.

In fact, none of the studies described above explains the reasons why both physical tests were not evaluated, despite the fact that it is widely known that when proposing a FPF battery, each of the components should be considered, which include, for example: morphological component, aerobic fitness, muscular strength, flexibility and motor agility/dynamic balance (25). This is explained by the fact that the establishment of normative values of the SFT with all its components provides a reference for OAs and health professionals to easily understand the measured levels. It even allows comparison between OAs and between populations from different regions and ethnicities (18), so the absence of both aerobic tests would imply important limitations in a test battery that evaluates the SFT.

We also highlight that the sample selection in six studies considered non-probability sampling and only one study considered probability selection. This study was developed in the Suzhuo region (China) (18). In fact, it is widely known that the probability sampling method is characterized by presenting the same chances of being selected in the sample (26, 27), whose results can be generalized to other realities or contexts. However, non-probability sampling, presents some limitations, for example, it does not guarantee equal opportunities for each subject in the target population (26) and consequently the results achieved cannot be generalized to other realities.

In essence, six out of seven studies have considered non-probabilistic sampling, so the references developed are limited to their own regions and countries. This opens up new possibilities for developing future studies with large sample sizes and especially with probability sample selection.

In relation to the second objective, this study aimed to describe the normative values expressed through percentiles of the SFT with its eight tests, according to age, sex and country. The seven studies have reported their percentile values at p10, p50 and p90, both in BMI and in the other tests: 30-s Arm curl, 30-s chair stand, chair sit-and-reach, Back scratch, 8-ft up-and-go, 2-min step test and 6-min walk.

In general, in relation to the six-minute walk test, the study by Rikli and Jones (3) carried out in the United States presents higher values in both sexes (in men ~70 to ~171 m and in women ~84 to ~125 m) compared to the other studies (4, 11, 13, 18–20). Although the study by Marques et al. (4) conducted in Portugal, evidenced a greater deterioration of aerobic fitness at older ages compared to their counterparts in Spain (three regions of China, Poland and United States).

In fact, a recent study conducted in Portugal, highlights the trends in functional fitness of older adults between 2008 and 2018. They highlight a trend toward a stabilization of most functional fitness tests during the last decade. However, they evidenced a critical decrease in the 6-min walk test (28), where they suggest urgently promoting a healthy aging process among Portuguese older adults.

In the biceps curl test, OA from the Hong Kong China region (20) presented poor performance in both sexes and in all age ranges relative to the other studies (3, 4, 11, 13, 18, 19). The six studies in general (two regions of China, Portugal, Spain, United States and Poland), presented similar results in both sexes and in all age ranges.

In the 30-s standing chair test (rep), the seven systematized studies present similar values in both sexes. However, the study conducted in United States (3) and Portugal (4), presented a rapid deterioration from 75 to 79 years of age onwards. Meanwhile, in the chair sitting-reach test (cm) and Back scratching (cm) all studies presented similar behaviors in both sexes and at all ages. Except for the study performed in Portugal by Marques et al. (4), in which a poor performance and rapid deterioration of flexibility at older ages is observed.

The values reported in the 8-ft up-and-go (sec) test in the seven studies are relatively similar and in both sexes. However, studies developed in Portugal (4) and Spain (11) present a rapid deterioration of agility with advancing age.

As for the 2-min step test (rep), six studies present similar values in all age ranges and in both sexes, although slightly, the study conducted by Chung et al. (20) in Hong Kong (China) evidences relatively lower values as age advances.

In sum, the seven systematized studies show in their percentiles deterioration of FF in both sexes as age increases. This decline is interpreted as a threat of loss of functional independence in OAs, as it is essential to maintain health-related fitness, which is achieved by regular long-term physical exercise and maintaining a healthy lifestyle (29).

SFT presents a great advantage due to its ability to estimate and track the rate of individual FF decline as a function of age and gender (3). In addition, normative SFT data allows for the assessment of individual performance, which helps to identify functional weaknesses in OA (20). This information serves to monitor and evaluate the motor domain of OA (19).

Based on the results achieved, this study facilitates the understanding of the functional fitness status of OAs from various geographical regions of the world. For it is widely known that cross-cultural comparisons in normative FPF values among OAs present differences in physical performance (4). These differences have to do with dynamic socio-economic changes, cultural, ethnic, genetic, geographic conditions very different between regions (5) as observed in this review.

In essence, the normative SFT data systematized in this study provide an ideal basis for constructing future curves in geographic regions where SFT tests have not been used or developed, so future studies should take our findings into consideration. In addition, normative data can serve not only to identify fitness levels, but also to promote public policies.

The study overall presents some strengths, given that it is the first systematic review study to focus on SFT percentiles in OA, it also describes each of the tests used in SFT in percentiles for both sexes. This information may be useful for researchers and practitioners working with OA. Notwithstanding the above, some possible limitations of the study have to do with the use of only two databases (Pubmed and Scopus). Future studies should include other databases to broaden the search spectrum and complement this study.

It is also necessary to suggest that researchers should be interested in developing studies of this nature, covering other geographic regions of the world (with probabilistic samples). As far as we know, there are few studies that cover large age ranges (65 to 95 years), for example, we did not find studies in Africa, South and Central America, leaving a clear gap in this area.

## Conclusion

In sum, this study concludes that from the first publication of the SFT to the year 2022, seven articles have been published in countries such as the United States, China, Poland, Portugal and Spain. However, no study has published the complete battery with its eight components. In addition, the normative FPF data in all systematized studies reflect decline with advancing age. The results of this systematization suggest its use for constructing new normative data, as well as for comparison with other regional and international studies.

## Data availability statement

The original contributions presented in the study are included in the article/supplementary material, further inquiries can be directed to the corresponding authors.

## Author contributions

MC-B: Conceptualization, Funding acquisition, Investigation, Methodology, Project administration, Writing – original draft, Writing – review & editing. RV-E: Investigation, Writing – original draft. IV-C: Writing – original draft, Data curation, Methodology. LC: Methodology, Writing – original draft, Investigation. ML: Investigation, Methodology, Writing – original draft, Formal analysis. CU-A: Formal analysis, Investigation, Methodology, Writing – original draft. JS-T: Investigation, Methodology, Writing – original draft, Writing – review & editing. RG-C: Investigation, Methodology, Writing – original draft, Writing – review & editing, Conceptualization, Formal analysis.

## Footnotes

^1^ https://pubmed.ncbi.nlm.nih.gov/
